# Medical education and abortion care: evaluating an interdisciplinary learning module in Germany

**DOI:** 10.1007/s00404-025-08269-z

**Published:** 2026-01-20

**Authors:** Kristina Killinger, Michelle Foerstel, Stephanie Wallwiener

**Affiliations:** 1 Department of Gynecology and Obstetrics, University Hospital Heidelberg, Im Neuenheimer Feld 440, 69120 Heidelberg, Germany; 2 Department of Obstetrics and Prenatal Medicine, University Hospital Halle (Saale), Halle, Germany

**Keywords:** Abortion education, Medical students, Reproductive health, Health policy, Attitude change

## Abstract

**Background:**

In Germany, first-trimester abortions are legally restricted but allowed under certain conditions, including mandatory counseling and a reflection period. Accessibility concerns persist. To address gaps in medical training, we developed an interdisciplinary learning module on first-trimester abortion care.

**Methods:**

We piloted the module in two sessions giving access to all medical students as an extracurricular learning opportunity. We conducted non-paired surveys across the medical school prior to the module and with our participants after the module to identify changes in attitudes as well as in intentions to treat.

**Results:**

We received a total of 297 responses. Most of the students (94%) were in favor of legalizing abortion laws. However, only 30% self-assessed their knowledge as sufficient, 40% of the students showed the willingness to perform abortions within the consultation clause and 43% of the students agreed to consult patients on abortion provision but not perform them themselves. The right for practitioners to object the performance of abortions was highly agreed upon (78%). After our pilot sessions, we received 53 evaluation surveys from 118 participants. Students reported a significant increase in knowledge. We observed a significant increase in general support and intention to treat after our module.

**Conclusions:**

Teaching about abortion is essential for our future healthcare providers. Overall, we see a great response to our new learning module and can hope for practice-changing effects on the provision of abortion care in the future. We integrated the module into our regular teaching catalogue.

## Introduction

In Germany, abortions within the first trimester are regulated through §218 of the German Penal Code (StGB) [1]. Women who seek to terminate their pregnancy penalty-free have to fulfill the following conditions: (1) intra-uterine pregnancy and gestational age diagnosed by a doctor via ultrasound; (2) compulsory counselling on the conflicting pregnancy by a state-approved center; (3) three-day reflection period; (4) abortion must be performed by a doctor and depending on the state even by a board-certified gynecologist [2, 3]. Abortions on request are not covered by statutory health insurance since abortions are regulated through the penal code [4]. The number of abortions registered in Germany has remained stable over the last 10 years with a count of around 100.000 abortions/year. Of these, 96% are performed upon request, 4% due to medical indications and 0,05% on criminological grounds [5]. Current data on regional access to abortion services is scarce in Germany [6]. Indirect indicators point out possible problems concerning availability and access. For example, the number of practices that registered abortions services decreased from approximately 2050 in 2003 to 1100 in 2022 [5]. There is also an increasing number of clients who turn to online abortion services [7] or travel to other European countries with simplified access [8]. The German government has taken steps to improve the situation. For instance, the German government dropped §219a from the German Penal Code (StGB), which forbid the advertisement of abortion services by providers to ameliorate transparency [9]. A recent nationwide study has shown that 80% of women with an unwanted pregnancy had non to little problems finding a doctor, who performs abortions [10].

In 2021, the new version of the nationally mandatory competence-based learning catalogue for medical students included theoretical aspects on abortion provision in Germany, but especially emphased on patient-provider communication tools [11]. In 2023, the first national guidelines on abortion provision in the first trimester was published by an expert commission [12]. With this background, we decided to create an interdisciplinary learning module on abortion provision in Germany.

Our theoretical framework for the learning module is based on the value clarification model, which is a process used in psychology and education to help the individual identify one’s core values. To affirm those values and contextualize them with social norms can help understand one’s behavioral intentions. It is important to state that value clarification does not divide into right or wrong but enhances the individual’s reflection process [13]. Value clarification workshops undertaken by IPAS, a nongovernmental organization within the field of sexual and reproductive health, have shown that through intensified reflection knowledge and attitudes concerning abortion care can change [14].

We designed a multi-stage learning module on ethical, socio-political and medical aspects of abortion provision within the first trimester in Germany. We consulted with ProFamilia and the ethics department for content and experts talks. The seminar was first piloted in May 2022, open to all medical students who subscribed, limited to 40 participants. It consisted of four submodules: (1) self-learning tool one week prior to the seminar; (2) three expert talks about ethical considerations, abortion provision and patient-centered communication, (3) role-playing exercises in small groups with actors to train patient-practitioner-interaction in the context of an unwanted pregnancy and (4) structured group discussions on legal, ethical, and practical aspects of abortion provision. After a pilot phase of two seminars, it was included into the mandatory education schedule.

To this point, little is known about the attitudes and intentions on abortion care of medical students in Germany. As they are the next generation of health care providers, gaining insight into their perspectives could help guide the development of effective training programs. Therefore, the aim of our study was to investigate the knowledge, attitudes and intentions of medical students in Germany concerning abortion care and whether our learning module had an effect, respectively.

## Methods

In May 2022, we conducted a self-administered online survey (Appendix A) using a modified version of the questionnaire developed by *Wheeler et. al (2012)* [15]. The survey was distributed through official medical school-wide mailing lists along with an explanation on the aim of the study. After two weeks, we closed the survey, right before starting our first pilot learning module. After three semesters, we conducted an anonymous second survey with all former participants from the pilot seminar and the mandatory module, including additional questions to evaluate the learning module. Individuals for the survey were recruited separately, firstly medical school wide to make general observations on the existing situation and demand for the module and secondly within the group of participants. There was no pairing between the two groups. Therefore, responses were treated as independent samples, and statistical analyses were conducted accordingly. This approach was chosen due to logistical reasons to allow comparison of group characteristics and outcomes without considering any within-subject relationship. The survey was written in German.

The survey consisted of four to five parts: socio-demographic variables, attitudes and beliefs about abortion provision in general, conditional attitudes and beliefs about abortion provision, future intentions to provide abortion care and for our participants of the learning module and an additional evaluation tool in the end.

Independent variables included age (grouped for < 18; 18–29; 30–44; 44–59 years), gender, relationship status (married; widowed; divorced; in separation; single; presently in a relationship), children (yes; no); religious affiliation (Christian; Jewish; Buddhist; Muslim; Hindu; other religious affiliation; not religious) and religious practice (regular; occasionally; never) and year in medical school (1–6 and > 6 years).

The survey consisted of 24 items majorly with Likert-scaled answers from “does not apply at all/do not agree at all” as 1 to “completely applies/completely agree” as 5. For descriptive statistics, we interpreted answers 1 + 2 as unsupportive 3 as ambiguous and 4 + 5 as supportive. Items were phrased in both supportive and unsupportive wording to ensure internal validity. Negative questions were reverse coded for statistical reasons. We summarized the survey answers according to their sections into (a) general support for abortion care, (b) conditional support for abortion care and (c) intention to treat as summary mean scores. When asking for reasons not to uptake abortion care we used a 4-point scale to form binary answers.

Firstly, we performed descriptive analysis where frequencies, proportions and means were tabulated. Differences between the two groups were examined using Mann–Whitney U Test for non-parametric, non-paired samples. Differences between more than two groups (e.g. different age groups) were determined using Kruskal–Wallis test for non-parametric, non-paired samples. For correlation analysis we used Spearman`s correlation coefficient for non-parametric distribution.

All statistical calculations were performed with Graphpad Prism, Version 10. Level of significance was set for *p* < 0,05. The study was approved by the ethics committee of the University of Heidelberg (S-616/2019).

## Results

Sample characteristics (Table 1).

**Table 1:** Sociodemographic characteristics of our study population services by group 1 (all medical students) and group 2 (attendees of the learning module)

		Group 1, total 297 *n* (%)	Group 2, total 53 *n* (%)
Year in Medical School	1	35 (11,8)	0
	2	83 (27,9)	0
	3	69 (23,2)	3 (5,6)
	4	51 (17,2)	9 (17,0)
	5	29 (9,8)	19 (35,9)
	6	15 (5,1)	16 (30,2)
	> 6	15 (5,1)	6 (11,3)
Age in years	< 18	1 (0,3)	0
	18–29	283 (95,3)	48 (90,6)
	30–44	13 (4,4)	5 (9,4)
	45–59	0	0
Gender	Female	217 (73,1)	39 (73,6)
	Male	79 (26,6)	13 (24,5)
	Nonbinary	1 (0,3)	1 (1,9)
Relationship status	Married	4 (1,3)	2 (3,8)
	Widowed	0	0
	Divorced	1 (0,3)	0
	Separated	3 (1,0)	0
	Single	152 (51,2)	23 (43,4)
	In a relationship	137 (46,1)	28 (52,8)
Participants with children	Yes	6 (2,0)	0
	No	291 (98,0)	53 (100)
Religion	Christian	122 (41,1)	23 (43,4)
	Jewish	1 (0,3)	0
	Buddhist	0	0
	Muslim	10 (3,4)	2 (3,8)
	Hindu	0	0
	Other	3 (1,0)	0
	Agnostic/Atheist	161 (54,2)	28 (52,8)
Religious practice	Non-practicing	208 (70)	39 (73,6)
	Unregularly practicing	68 (22,9)	13 (24,5)
	Regularly practicing	21 (7,1)	1 (1,9)

We received 297 responses from our first medical school-wide survey, which accounts for a response rate of 14,9%, with around 2000 medical students being enrolled in the program at the University of Heidelberg. From 118 students attending the learning module from summer semester 2022 until winter semester 2023, we received 53 answers to our survey and evaluation. This represents a response rate of 44,9% for our second survey.

Almost all our respondents in both groups were between 18 and 29 years old (95,3% and 90,6%, respectively). While the first group was mixed concerning their progress in medical school with the highest proportion of participants being in their third year, the second group consisted of students, who were at least in their third year of medical school. This is explained by having gynecology modules more to the end of medical school in general. Around 73% of participants identified as female, around 26% as male and 1% as non-binary. In the first group, slightly more than half of the participants were single (51,2%) compared to around 43% in the second group. Here, more than half of the participants were in a relationship (52,8%). While in the first group 6 participants reported to already have children, none of our participants in the second group already had children. In both groups, more than half of respondents identified as agnostic/atheist (54,2% and 52,8%, respectively). Christians represented 41,1% and 43,4%, constituting the largest religious group followed by Muslims with 3,4% and 3,8%. When asked about religious practice, most reported to be non-practicing (70% and 73,6%, respectively), some reported to be irregularly practicing (22,9% and 24,5%, respectively) and very few reported to be regularly practicing (7,1% and 1,9%, respectively). Both samples showed similar compositions in the above-named characteristics (Table 1).

Support for abortion provision (Table 2).

**Table 2:** Attitudes, conditional support, knowledge and intentions to act on abortion services by group 1 (all medical students) and group 2 (attendees of the learning module)

Statements (*n* = Group 1/Group 2)	Group 1 mean likert scale (*R* = 1–5) pro—neutral—contra in %	Group 2 mean likert scale (*R* = 1–5) pro—neutral—contra in %	*p* (two-sided)
**General attitude and beliefs**			
Abortions should be legal and accessible for everyone (*n* = 297/53)	4,7	4,8	0,381
	93,6–3,0–3,4	100–0–0	
Abortions should be part of the statutory health insurance benefits (*n* = 296/53)	4,4	4,5	0,277
	85,8 -10,5–3,7	69,8–17,0–13,2	
I find abortions to be morally reprehensible (*n* = 295/53)	1,7	1,4	0,062
	7,8–7,5–84,7	3,8–3,8–92,4	
Abortions should not be provided in any case (*n* = 297/53)	1,1	1,0	0,059
	1,4–1,0–97,6	0–0–100	
A woman should have the right to decide for herself whether to have an abortion (*n* = 297/53)	4,7	5,0	0,135
	94,9–2,4–2,7	100–0–0	
**Conditional support for abortion provision**			
Abortions should be legal if the woman's health is endangered by the pregnancy. (*n* = 297/53)	4,9	5,0	0,176
	98,7–0,3–1,0	100–0–0	
Abortions should be legal if the woman's mental well-being is endangered by the pregnancy. (*n* = 297/53)	4,8	4,9	0,196
	94,9–2,7–2,4	98,1–1,9–0	
Abortions should be legal if the mother or family cannot afford a child financially. (*n* = 296/53)	4,1	4,3	0,697
	73,7–13,5–12,8	79,2–17–3,8	
Abortions should be legal if the fetus shows signs of a serious genetic disease or malformation. (*n* = 297/53)	4,4	4,5	0,312
	80,8–13,1–6,1	84,9–11,3–3,8	
Abortions should be legal if the pregnancy is the result of rape. (*n* = 296/53)	4,9	4,9	0,299
	96,6–1,7 -1,7	100–0–0	
Abortions should be legal on demand regardless of gestational age. (*n* = 294/53)	3,6	3,1	0,033*
	55,4–17,4–27,2	39,6–22,6–37,8	
**Knowledge and intentions to act**			
I have sufficient knowledge of the medical and legal basics of abortion. (*n* = 293/52)	2,9	3,9	< 0,001*
	32,8–28,7–38,5	71,2–23,1–5,7	
I would like to perform abortions within the consultation clause as soon as I am trained for it. (*n* = 294/52)	3,1	3,3	0,309
	40,5–27,9–31,6	50,0- -25,0–25,0	
In the future, I would like to consult patients on pregnancy conflicts with an open mind but refer them to others for abortion. (*n* = 290/52)	3,2	3,4	0,244
	43,1–26,9–30,0	46,2–26,9–26,9	
Under no circumstances do I want to consult patients on pregnancy conflicts. (*n* = 291/52)	1,3	1,2	0,132
	2,4–3,4–94,2	1,9–5,8–92,3	
Under no circumstances do I want to perform abortions in the future, regardless of the indication. (*n* = 288/52)	1,9	1,9	0,781
	13,5–11,8–74,7	11,5–11,5–77,0	
I do not want to perform abortions within the consultation clause in the future. (*n* = 287/52)	2,1	1,8	0,119
	15,7–15,7–68,6	7,7–15,4–76,9	
In the case of pregnancy conflict counseling, I would try to convince the patient to carry out the pregnancy. (*n* = 288/52)	2,1	1,5	< 0,001*
	11,1–26,0–62,9	3,8–7,7–88,5	
Doctors should be able to decide freely whether to perform abortions. (*n* = 293/52)	4,2	4,2	0,663
	78,5–9,9–11,6	77,0–11,5–11,5	

We observed positive attitudes towards abortion seekers and abortion provision overall. In the general attitudes and beliefs section both groups expressed support in over 85%. Although there were no significant differences overall, a slight trend towards firmer beliefs in the second group could be observed within the negative statements: I find abortions to be morally reprehensible (*p* = 0,062) and Abortions should not be provided in any case (*p* = 0,059). The general liberal stance on abortion provision continued in the conditional support section. Here, we did observe high support rates and did not observe any major differences with one exception. When asked about support regardless of gestational age, the second group that had undergone the learning module showed significantly more ambiguity and less support with 39,6% in group 2 vs. 55,4% in group 1 (*p* = 0,033). We observed a significant boost in self-reported knowledge in the second group (*p* < 0,001). Importantly, we observed liberal attitudes and beliefs regarding abortion in both groups, but a much higher ambiguity when it came to the participants’ intentions to actually perform abortions. Around 40% (group 1) and 50% (group 2) of respondents wanted to perform abortions in the future and around 43% (group 1) and 46% (group 2) wanted to consult patients but refer for an abortion. The only significant difference between the group was seen in the negative statement: In the case of pregnancy conflict counseling, I would try to convince the patient to carry out the pregnancy (*p* < 0,001). Here, the second group showed firmer rejection to the statement. Both groups affirmed the statement that doctors should be able to choose freely whether they would like to perform abortions.

**Fig. 1:**
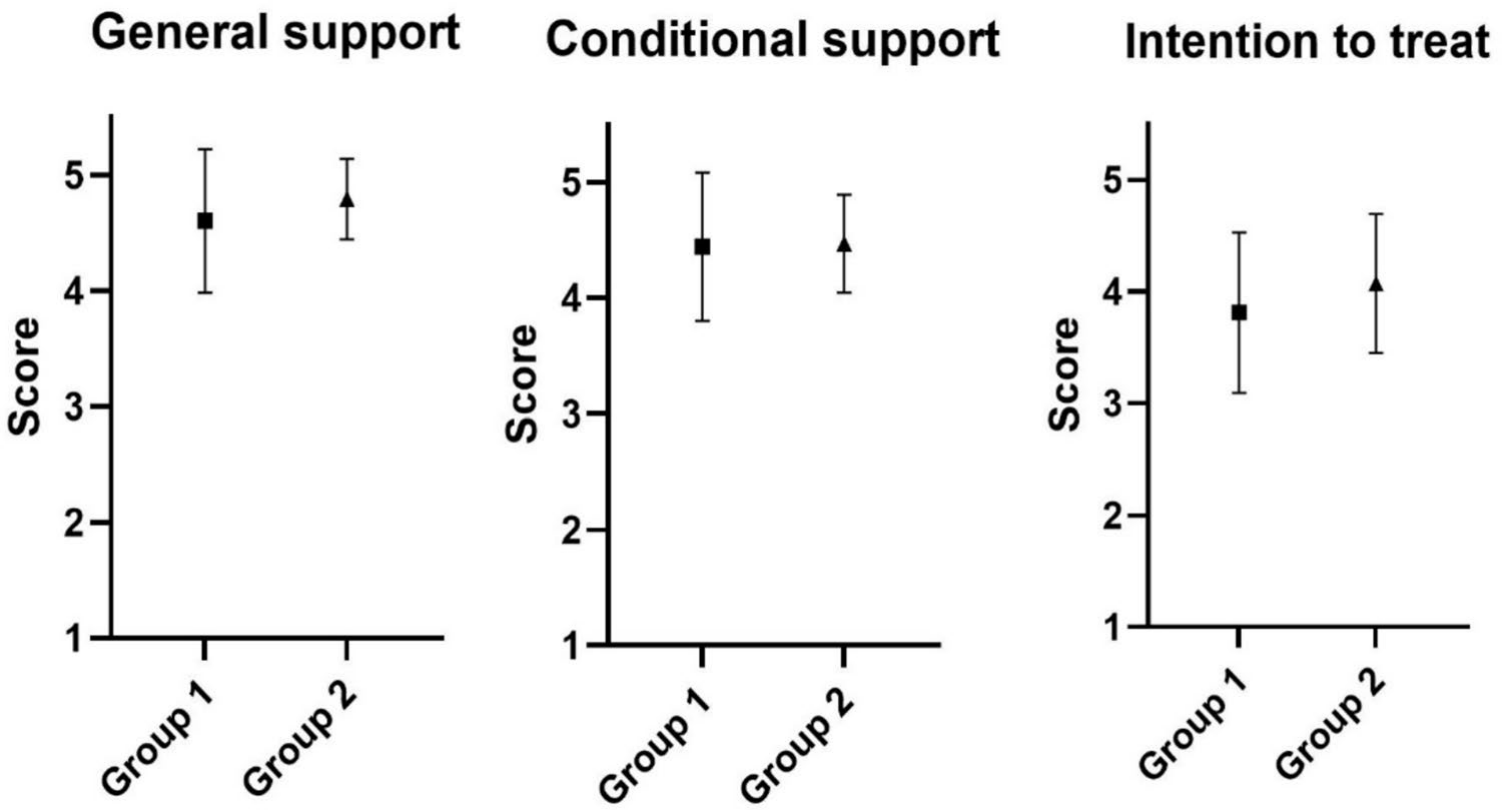
Comparison of the two groups concerning their general support, conditional support and intention to treat in abortion care; Group 1 = all medical students; Group 2 = students who attended the module

We compared the mean summary scores between both groups and found a significant rise in the general support for abortion provision (*p* = 0,0418) as well as in the intentions to treat (*p* = 0,0107). Solely conditional support for abortion provision did not change significantly (*p* = 0,4384) (Fig. 1).

**Fig. 2:**
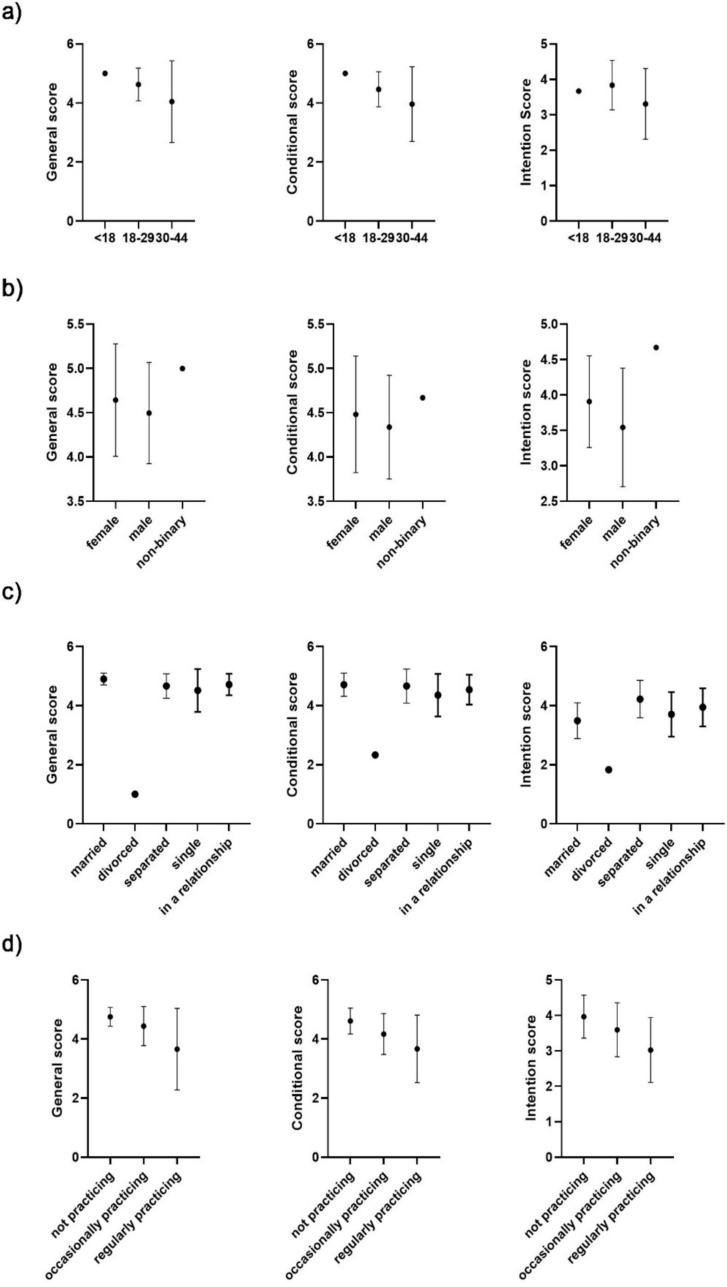
Comparison of different socio-demographic factors in correlation with general support, conditional support and intention to treat scores; **a** age, **b** gender, **c** relationship status and **d** religious practice

Differences in support depending on socio-demographics (Fig. 2).

When examining socio-demographics and their influence on mean summary scores for general and conditional support as well as intention to treat, we analyzed our first survey data with answers from 297 medical students. We excluded the independent variable of having children. Due to an insufficient number of events in this group, a meaningful comparison could not be made. We also excluded religious affiliation for the same reason but used the surrogate item of religious practice. We found no significant difference in mean summary scores concerning our three age groups (general support *p* = 0,0610; conditional support *p* = 0,3170; intention to treat *p* = 0,1122). On the other hand, we did see a significant difference in mean summary scores between gender groups with the female group showing higher support and intention to treat (general support *p* = 0,0067; conditional support *p* = 0,0351; intention to treat *p* = 0,0006). Concerning relationship status there were no significant differences in support, but in intention to treat (general support *p* = 0,0687; conditional support *p* = 0,0759; intention to treat *p* = 0,0085). Religious practice was associated with lower support and intention to treat (general support *p* < 0,0001; conditional support *p* < 0,0001; intention to treat *p* < 0,0001).

**Fig. 3:**
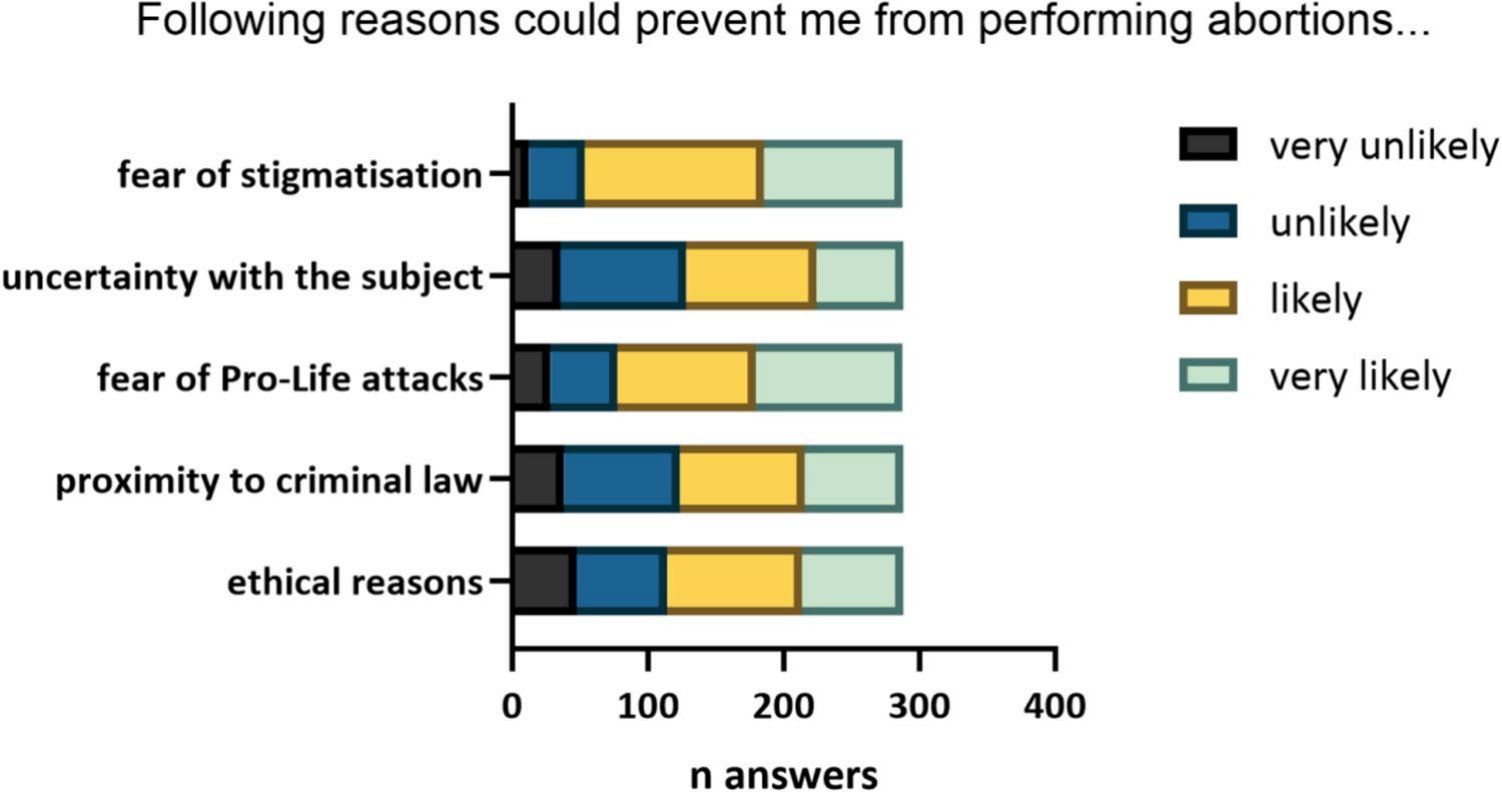
Distribution of answers given for possible reasons that could prevent students from performing abortion care on a scale from 1–4 (very unlikely-very likely)

When asked about possible reasons for not performing abortion care the fear of stigmatization and possible pro-life attacks stood out from the other provided answers (ethical reasons, proximity to criminal law, uncertainty with the subject) (*p* < 0,0001) (Fig. 3).

## Discussion

While the legality of abortion care is a constant topic of discussion within the German Congress, it is important to look at future medical doctors and how they perceive abortion care, as well as their intention to treat and consult patients with pregnancy conflicts. Compared to similar studies, we observed extremely high rates of support among our student population. The study by *Wheeler *et al*.* [15] from which we derived our survey showed that 40% of students in South Africa thought that abortion should be legal compared to ca. 93% in our student group. Similar high support was observed among students in Canada and the United Kingdom [16, 17]. In all these countries, abortions are available upon request, but barriers differ due to a lack of providers. Looking at our future generation of potential providers is therefore essential. Like in prior studies, we see that positive attitudes towards abortion care (93%) do not necessarily translate to high intention to treat (40%) in the future. We also see that gender, relationship status and religious practice play an important role concerning the willingness to provide abortion care in the future. A significant proportion of our students cited fear of stigmatization and fear of pro-life attacks as reasons for potentially avoiding abortion care in the future. This differs from findings among U.S. medical students, who frequently reported personal and religious beliefs and a lack of training as primary reasons for not providing abortion care [18]. Even though our students did not explicitly name ethical reasons, we saw a significant decrease in support with more religious practice. Whereas religious norms and ethical reasons are intrinsic factors, the fear of stigmatization, pro-life attacks and a lack of training are barriers that can be addressed. Interestingly, in a UK survey it could be shown that students rated teaching in this field as important no matter their personal views on the subject. They also wished for more simulated trainings on speaking to patients requesting abortions [17]. As our theoretical framework, we used the values clarification framework by *Turner *et al. [14] aiming to help our students clarify what their values are, what ethical reasoning for liberal vs. conservative views exist and which communication techniques can be used when confronted with abortion-seeking in their clinical practice. While our goal was not to change students’ attitudes or shift their values, we observed significantly more general support for abortion care as well as more intention to treat in the group that completed the learning module. At the same time, students showed more ambiguity towards providing abortion care regardless of gestational age. This finding might hint towards more reserved views towards late abortions. This fits well with similar findings in another study conducted by doctors for choice [19]. Students estimated their knowledge in the field as significantly higher after the learning module. This suggests a deeper engagement with the topic, as second-trimester abortions are often more complex both medically and psychologically for providers and patients compared to first-trimester procedures [20]. However, as our survey did not explicitly examine attitudes toward second-trimester abortion support, this interpretation remains speculative.

## Limitations

The major limitation lies within the study design. We surveyed the two groups independently without pairing the answers. This design was chosen due to logistic reasons and trying to have an extremely low threshold to answer the questionnaire within the medical school population. We acknowledge, without paired responses and due to disparities between the two groups, it becomes challenging to establish causal relationships on behalf of the intervention, as we cannot directly compare before-and-after-effects. We still estimate that our learning module left an impression since both general support as well as intention to treat as well as knowledge in the matter were significantly higher in the group that attended the learning module. We acknowledge that these effects were measured only once, and the elapsed time between the module and the survey may have led to lower participation rates and potentially attenuated the observed effects. Another major limitation is self-selection bias due to the nature of the online survey. When participants choose themselves to be part of the survey, we must assume overrepresentation of students who are more interested in the subject and, therefore, might have more liberal attitudes. This might also be seen for the socio-demographics of our study population, since we know that attitudes in metropolitan areas within student groups are often more liberal compared to rural areas [21]. Further research on pre-post-effects is needed and could be within the scope of future multi-centric efforts to incorporate the module within the medical curriculum. Additionally, surveys on a nation-wide level are necessary to really make representative statements on the attitudes and intentions to treat of our future abortion care providers in Germany.

## Conclusion

The majority of medical students in this study endorsed the legality and accessibility of abortion. However, a relatively smaller number indicated a willingness to personally provide abortion services in their future medical careers. The students in our study clearly represent a motivated group actively seeking additional education on abortion. Hence, our findings are not generalizable to the entirety of medical students in Germany. However, evidence suggests that clinical exposure to abortion training during medical school can positively impact a broader array of students. We therefore advocate for a broad implementation of educational modules concerning abortion care and family planning in Germany.

## Data Availability

The data that support the findings of this study are available from the corresponding author upon reasonable request. Due to ethical considerations and participant confidentiality, data are not publicly available. Any requests for access to the anonymized datasets will be reviewed and granted on a case-by-case basis in accordance with institutional and legal guidelines.
